# Vitamin D_3_ Supplementation and Antibiotic Consumption – Results from a Prospective, Observational Study at an Immune-Deficiency Unit in Sweden

**DOI:** 10.1371/journal.pone.0163451

**Published:** 2016-09-22

**Authors:** Anna-Carin Norlin, Susanne Hansen, Emilie Wahren-Borgström, Carl Granert, Linda Björkhem-Bergman, Peter Bergman

**Affiliations:** 1 Infectious Disease Clinic, Karolinska University Hospital, SE-141 86 Stockholm, Sweden; 2 Division of Clinical Immunology, Department of Laboratory Medicine, Karolinska Institutet, 141 86, Stockholm, Sweden; 3 Division of Clinical Microbiology, Department of Laboratory Medicine, Karolinska Institutet, 141 86 Stockholm, Sweden; Medizinische Universitat Graz, AUSTRIA

## Abstract

**Background:**

Vitamin D supplementation has been proposed to improve clinical symptoms during respiratory tract infections (RTIs), but results from randomized, placebo-controlled trials (RCT) are inconclusive. Previously, we performed an RCT in patients with various immune-disorders and observed that supplementation with 4000 IU vitamin D/day during 12 months significantly reduced antibiotic consumption and RTIs. This formed the basis for new guidelines at our unit; i.e. patients with insufficient levels of 25-hydroxyvitamin D (≤75 nmol/L) are now offered vitamin D supplementation. The aim of this prospective follow-up study was to evaluate the outcome of these new recommendations with regard to antibiotic consumption in our unit.

**Method:**

277 patients with insufficiency were supplemented with vitamin D_3_, 1500–1600 IU/day for 12 months. Each patient was its own control and data on antibiotic consumption was monitored 12 months before and 12 months after initiation of vitamin D_3_ supplementation.

**Results:**

Vitamin D_3_ supplementation resulted in a significantly reduced antibiotic consumption, from 20 to 15 days/patient (p<0.05). The number of antibiotic-free patients increased from 52 to 81 after vitamin D_3_ supplementation; OR 1.79; 95% CI 1.20–2.66 (p<0.01). The number of antibiotic-prescriptions decreased significantly, a finding that mainly was attributed to a reduction of respiratory tract antibiotics (p<0.05). Subgroup analysis showed that only patients without immunoglobulin substitution (n = 135) had a significant effect of vitamin D supplementation.

**Conclusion:**

Vitamin D_3_ supplementation of 1600 IE /day is safe to use in immunodeficient patients with 25-OHD levels less than 75 nmol/L and significantly reduced the antibiotic consumption in patients without immunoglobulin substitution.

## Introduction

Vitamin D is important for a healthy immune system and plays an important role in innate immunity by inducing synthesis of antimicrobial peptides [1]. In addition, vitamin D has broad anti-inflammatory effects on the adaptive immune system [2, 3]. We have previously conducted a randomized, placebo-controlled and double blind study where patients with frequent respiratory tract infections (RTIs) followed at the Immunodeficiency Unit at Karolinska University Hospital, were randomized to placebo or vitamin D_3_ (4000 IU/day for 1 year). In this study (n = 124, *per protocol*) we could show that vitamin D_3_ treated patients (n = 62) had significantly reduced infectious symptoms, measured as “infectious score”, and a 60% reduction of antibiotic consumption compared to the placebo group (n = 62) [4]. In a post-hoc analysis we showed that vitamin D_3_ supplementation increased the probability to stay free of RTIs during the study year and that the total number of RTIs was reduced in the vitamin D_3_-group [5]. Furthermore, the time to the first RTI was significantly extended in the vitamin D-group [5]. Interestingly, patients who reached 25-hydroxyvitamin D-levels (25OHD) > 100 nmol/L at the study end, generally had a better well-being than patients with lower vitamin D levels [6].

Several randomized and placebo-controlled trials have been performed studying vitamin D supplementation and RTIs [4, 7–17]. In a meta-analysis of 11 such studies, including 5660 patients, we found that vitamin D_3_ had a protective effect against RTIs and that dosing once-daily seemed most effective [18]. At the Immunodeficiency Unit we have incorporated analysis of serum level of 25OHD and initiate supplementation with 1500–1600 IU/day to those with 25OHD-levels below 75 nmol/L, which in previous studies have been shown to be the critical threshold level for protection against RTI [19, 20]. In fact, many of the patients who took part in the previously mentioned RCT are still visiting our clinic and in light of the positive results from that trial, it was a logical consequence to perform a follow-up study in clinical practice.

Therefore, we designed a study to assess whether such a new clinical paradigm would lead to any benefit for the individual patient. To obtain non-biased data, we decided to collect information on antibiotic prescriptions from the national pharmaceutical registry, where all prescriptions in Sweden are registered.

## Material and Methods

### Ethical statement

The study was approved by the regional Ethical Review Board at Karolinska Institutet, Stockholm, Sweden (dnr 2013/2244-31/1) and an approval was also obtained from the Swedish Medical Product Agency that the study fulfilled the criteria for a “non-interventional clinical trial.” Written informed consent was obtained from all participants before inclusion and the study was conducted in accordance with the Declaration of Helsinki.

### Study design

**Fig 1:**
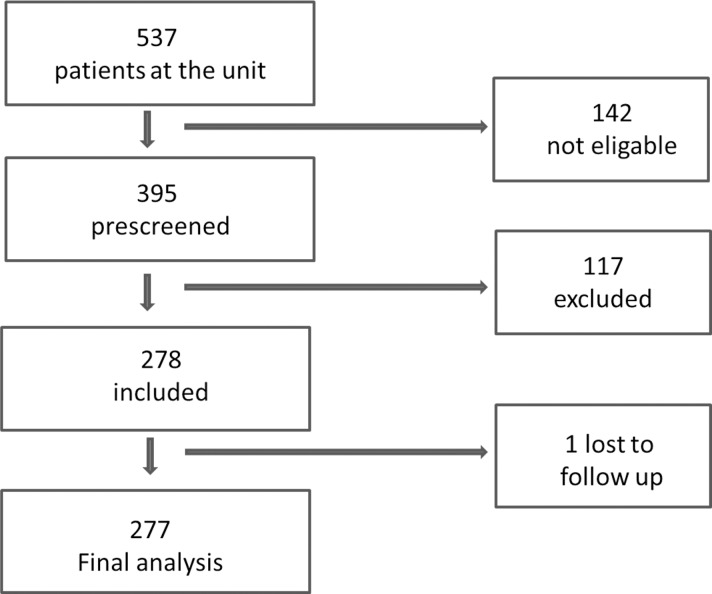
Flowchart of included patients. 142 patients were not screened due to exclusion criteria (sarcoidoses, tuberculosis or already on vitamin D treatment) or if they were not planned to be followed at the clinic for at least one year or if they for other reasons were considered not suitable for participating in a trial. 111 were excluded due to 25 OH D-levels above 75 nmol/L and 6 declined to participate (excluded n = 117). One patient died during the study period (lost to follow up) and was therefore excluded from the final analysis.

Patients were recruited from the Immunodeficiency Unit, Infectious Disease Clinic, Karolinska University Hospital, Stockholm, Sweden, between March 2013 to October 2013. The immunodeficiency Unit is an outpatient clinic and a tertiary referral unit for taking care of patients with an increased susceptibility to infections. In total approximately 1000 patients are registered at the unit with a majority having IgG-deficiency. Inclusion criteria were 25OHD-levels of ≤75 nmol/L. Exclusion criteria were sarcoidosis, tuberculosis or treatment with cholecalciferol or with active vitamin D (Etalpha). A flowchart of included patients is shown in Fig 1. N = 537 patients were visiting the Immunodeficiency Unit during this time and n = 395 were pre-screened for participating in the study. N = 142 patients were not screened due to exclusion criteria or if they were not planned to be followed at the clinic for at least one year or if they for other reasons were not considered suitable for participating in a trial (see Fig 1, not eligible n = 142). Among the n = 395 screened patients n = 111 were excluded due to 25OHD-levels above 75 nmol/L and n = 6 declined to participate (see Fig 1, exclusion n = 117). N = 278 patients were included in the study and n = 1 patient died during the study period (see Fig 1, lost to follow up n = 1). Finally, n = 277 patients could be included in the final analysis.

Patients were given either cholecalciferol (Vitamin D_3_) as two tablets of Divisun 800 IU/tablet i.e. 1600 IU/day or 3 drops of Detremin-oil 500 IU/drop i.e. 1500 IU/day. Every other patient included in the study was prescribed Detremin and every other Divisun. This procedure was chosen to use the two cholecalciferol-products available in the Swedish market that are produced according to good manufacturing practice (GMP) and therefore possible to prescribe in Swedish pharmacies.

The patients had between 1–4 visits to the Immunodeficiency Unit during the study year. The average time point for the first follow-up visit was 6 months after starting vitamin D treatment but the time point differed between 3 months up to 12 months. At each visit patients were asked about compliance, which was noted in the CRF. Data on serum levels of 25OHD was recorded and filled out in the CRF. Plasma levels of calcium were also obtained for safety reasons. No other blood chemistry parameters were collected as a part of this study.

### Antibiotic prescription

Data on antibiotic prescription for each patient was retrieved from the Swedish Prescribed Drug Registry, where all prescribed drugs that have been bought in a pharmacy in Sweden are registered. The main focus here has been on per orally taken antibiotics and therefore colistin (1 prescription) and tobramycin (3 prescriptions), which are used for inhalation, are not included in the final analysis despite that this information was retrieved from the registry. Antibiotic consumption for each patient was determined for the year before and for the year after initiation of vitamin D_3_ supplementation. Antibiotic consumption was expressed as ‘number of days with antibiotics for 12 months’ and also as ‘number of prescriptions for 12 months’. In addition, ‘type of prescribed antibiotics’ was collected. 25OHD-levels and ionized calcium were measured regularly after starting on vitamin D_3_ supplementation.

An analysis where the antibiotics were divided in to “respiratory tract antibiotics” and “other antibiotics” was also performed. The definition of “respiratory tract antibiotics” as recommended from the Swedish Strategy Group for Rationale use of Antibiotics (STRAMA) (www.strama.se) was used in the analysis, with the exception of ceftibuten, which is not used for respiratory tract infections at the Karolinska University Hospital (local recommendations). The STRAMA-definition is used for all official statistics on antibiotic usage in Sweden and in various comparisons with other countries.

### Statistical analysis

Statistical analyses were performed using Graph Pad Prism vs 6.0. Since the data did not show Gaussian distribution, non-parametric tests were used and in the tables and figures median values and interquartile ranges are shown. In the text the whole range (min-max) are stated for age and 25OHD levels. For comparison between groups, Wilcoxon matched-pairs signed rank test was used. For analysis of the number of patients with or without antibiotics the year before or after inclusion, Fishers exact test was used. For comparison between differences in prescription of respiratory tract antibiotics compared to “non-respiratory” antibiotics, unpaired t- test was used since these data were normally distributed.

## Results

### Baseline demography and 25-hydroxyvitamin D levels

Two hundred seventy seven patients (n = 277) were included in the study and followed for 1 year. The median age of the cohort was 55 years (range 18–90) and there were 175 women and 102 men. The patients included had different diagnoses, where various IgG-deficiencies were predominant, as presented in Table 1. Of the 277 patients 135 (49%) had treatment with immunglobulins during the study period (Table 1). Ninety-seven patients (35%) had at least one diagnosis of respiratory disease; 45 (16%) had asthma, 36 had COPD (13%) and 16 (6%) had other respiratory diseases (bronchiectasis, lung embolus, lung cancer, chronic sinusitis, chronic bronchitis, lung sarcoidosis, respiratory insufficiency). The median 25OHD-level of the cohort was 54 nmol/L (range 10–75) at inclusion, for both women and men. After 1 year of supplementation the median value was 86 nmol/L (Table 2), 88 nmol/L for women and 78 nmol/L for men. Fourteen of the 277 patients (5%) had no increase or a slight decrease in 25 OHD levels, whereas a majority, 95%, exhibited raised 25OHD-levels, as a sign of compliance to the prescription.

**Table 1:** Diagnoses in the study cohort. CVID, Common Variable Immuno-Deficiency. ‘IgG Suppl’, number of patients with Immunoglobulin supplementation.

	Number of patients (n = 277)	IgG. Suppl. (n = 135)
Selective IgA deficiency	44	4
IgG subclass deficiency	80	34
CVID	52	52
Increased susceptibility to infections	72	28
T-cells disorders	2	1
Di George Syndrome	1	1
Hyper IgE syndrome	2	1
IgA relative deficiency	3	2
Complement deficiency	4	2
Secondary antibody deficiency	7	3
WHIM syndrome	1	1
X-linked antibody deficiency	5	5
Unknown	4	1

**Table 2:** Antibiotic consumption and 25-hydrovitamin D levels in the whole study cohort and in the different subgroups before and after 1 year of vitamin D_3_ supplementation.

	Before vitamin D_3_ supplementation	After vitamin D_3_ supplementation	p-value
All (n = 277)			
25-OH vit D (nmol/L)	54 (42–64)	86 (70–100)	p<0.001
Number of prescription	2 (1–5)	2 (0–4)	p<0.01
Days of antibiotics/year	20 (8.5–47)	15 (0–40)	p<0.01
Subgroup < 30 nmol/L (n = 29)			
Number of prescription	1 (0–4.5)	2 (0–5)	ns
Days of antibiotics/year	10 (0–43)	13 (0–44)	ns
Subgroup 30–50 nmol/L (n = 86)			
Number of prescription	3 (1–6)	2 (1–5)	ns
Days of antibiotics/year	27 (10–51)	19 (7–48)	ns
Subgroup >50 nmol/L (n = 162)			
Number of prescription	2 (1–4)	1 (0–4)	p<0.01
Days of antibiotics/year	20 (9–41)	10 (0–37)	p<0.05
Subgroup Ig suppl (n = 135)			
Number of prescription	3 (1–6)	3 (1–5)	ns
Days of antibiotics/year	27 (10–56)	26 (9–53)	ns
Subgroup Non Ig suppl (n = 142)			
Number of prescription	2 (1–3)	1 (0–2)	P<0.01
Days of antibiotics/year	17 (5–37)	10 (0–24)	p<0.05

Values show median and interquartile range within parenthesis. P-values are calculated by using Wilcoxon matched-pairs signed rank test. ns = non significant.

To investigate if subjects with low 25OH D-levels would benefit more from vitamin D_3_ supplementation compared to subjects with higher levels, the cohort were divided into three groups based on the vitamin D levels at inclusion: 25OHD-levels < 30 nmol/L, 30–50 nmol/L and >50 nmol/L and subgroup-analyses were performed. The median 25OHD-levels in the different subgroups are shown in Table 2.

### Antibiotic consumption

**Fig 2:**
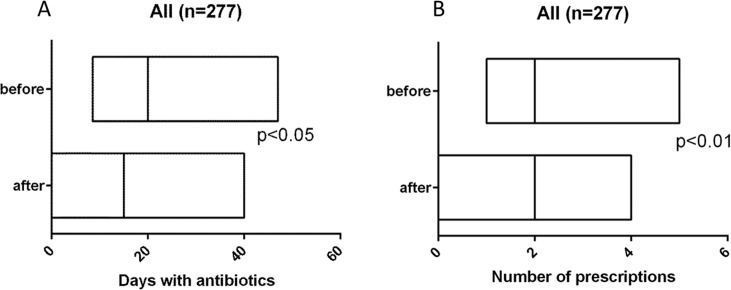
Number of days with antibiotics (A) and number of prescriptions (B) per patients the year before and after starting on vitamin D treament. The lines shows median and boxes show interquartile range. Statistical test was performed using Wilcoxon matched-pairs signed rank test.

Days of antibiotic supplementation were registered for each patient for the period of 12 months before supplementation and during 12 months after start of supplementation, as shown in Fig 2A. The year before supplementation the median number of days with antibiotics per patient was 20 (interquartile range; 8.5–47) and the year during vitamin D_3_ supplementation the number decreased to 15 days (interquartile range; 0–40), p<0.05). In addition, the number of antibiotic prescriptions per patient decreased significantly during vitamin D_3_ supplementation compared to the year before, as shown in Fig 2B. The year before supplementation the interquartile range of the number of prescriptions was 1–5, and the year during vitamin D_3_ supplementation the interquartile range was 0–4 (p<0.01).

**Fig 3:**
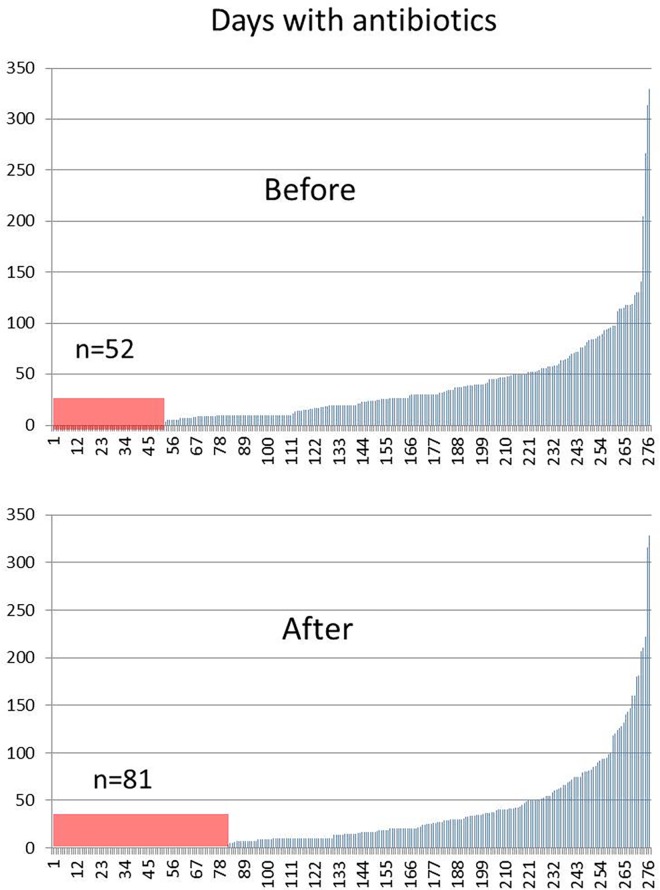
Days with antibiotics for each patient (n = 277) the year before and after starting on vitamin D treament in the study cohort. Vitamin D supplementation resulted in more patients that could be without antibiotics during the year, the number of patients with no antibiotics increased from 52 to 81; Fischer exact test showed OR 1.79; 95% CI 1.20–2.66 (p<0.01).

Vitamin D_3_ supplementation resulted in significantly more antibiotic-free patients during the year after supplementation; the number of antibiotic-free patients increased from 52 to 81; OR 1.79; 95% CI 1.20–2.66 (p<0.01) as shown in Fig 3.

## Subgroup analysis

Interestingly, the subgroup analysis for the different vitamin D levels at inclusion, showed that the patients with lowest levels at inclusion, 25OHD-levels < 30 nmol/L, had less benefit of vitamin D_3_ supplementation than patients that had 25OHD-levels > 50 nmol/L (Table 3). Patients with immunoglobulin (Ig) supplementation had a higher consumption of antibiotics and had no significant beneficial effect of vitamin D supplementation (Table 2). In contrast, the non Ig supplemented group had a positive effect of vitamin D treatment with a significant decrease in antibiotic consumption (Table 2). Patients with respiratory disease (n = 97) generally had more days with antibiotics than the rest of the study cohort but the beneficial effect of vitamin D treatment did not differ significantly from the rest of the cohort (data not shown). Further subgroup analysis showed that vitamin D_3_ supplementation was beneficial for both men and women to the same extent (data not shown).

**Table 3:** Type of Antibiotics prescribed the year before and after starting vitamin D supplementation.

	Type of Antibiotics	Prescriptions Before (n)	Prescriptions After (n)	Difference
	**Respiratory tract antibiotics**^**1**^			
1	Amoxicillin	225	199	-26
2	Cefadroxil	18	5	-13
3	Doxycycline, Tetracycline	195	141	-54
4	Erythromycin, Clarithromycin, Azithromycin	56	47	-9
5	Phenoxymethylpenicillin	100	67	-33
			**Summary:**	**-135**
	**Other antibiotics**			
	*Staphylococcal antibiotics*			
6	Flucloxacillin	53	49	-4
7	Clindamycin	18	38	+20
	*Quinolones*			
8	Ciprofloxacin, Levofloxacin, Moxifloxacin	98	134	+36
	*Urinary tract antibiotics*			
9	Trimethoprim	3	4	+1
10	Nitrofurantoin	23	14	-9
11	Pivmecillinam	32	18	-14
12	Ceftibuten^2^	2	20	+18
	Mixed indications			
13	Trimethoprim-sulfamethoxazole	43	37	-6
14	Rifampicin	2	0	-2
15	Lymecycline^3^	1	5	+4
			**Summary:**	**+44**
	**Summary all:**	**869**	**778**	**-91**

^1^Defines as antibiotics used mainly for treating respiratory tract infections according to Swedish Strategy Group for Rationale use of Antibiotics (www.strama.se).

^2^Ceftibuten is strictly used for urinary tract infections according to local clinical recommendations.

^3^Belongs to the tetracycline family, but is strictly used for treatment of acne vulgaris.

### Type of antibiotics

Since it is possible that vitamin D_3_ supplementation is more beneficial against RTIs than against other infections [21, 22], the changes in antibiotic prescription in relation to indication were analyzed. A summary of all antibiotics prescribed in the study is presented in Table 3. Notably, the prescriptions of antibiotics to treat RTIs, defined according to STRAMA with the exception of ceftibuten, decreased significantly, mean value 2.14 prescriptions/patient the year before to 1.66 prescriptions/patient for the year after start of vitamin D_3_ supplementation (minus 0.48 prescriptions/patient). In contrast, the prescriptions of non-respiratory antibiotics increased slightly during the study period; 0.99 prescriptions/patient before and 1.15 prescriptions/patient after vitamin D_3_ supplementation (+0.16 prescriptions/patient, p = 0.003, student’s t-test, Table 3).

### The general antibiotic prescription pattern at the Immunodeficiency Unit

To obtain a measure of the general antibiotic prescription pattern at the Immunodeficiency Unit during the study period, we retrieved information on all prescriptions during 2012, 2013 and 2014 (36 months) from the Strategic Programme Against Antibiotic Resistance (STRAMA), that registers all antibiotic prescriptions in Sweden (Annika Hahlin, STRAMA, personal communication). Before the study started (January 2012-February 2013), there were 38 prescriptions of respiratory antibiotics per month. After the inclusion was completed (November 2013-December 2014), there were 40 prescriptions per month of respiratory antibiotics. Importantly, these figures indicate that there was no general decrease in the prescription patterns during the study period, rather the numbers increased slightly. For non-respiratory antibiotics, the figures were 17 prescriptions per month before (Jan 2012-Feb 2013) and 22 per months after (Nov 2013-Dec 2014).

### Adverse events

There were no serious adverse events reported during the study. The vitamin D_3_ supplementation was generally well tolerated. One patient taking Divisun experienced nausea and diarrhea, but this disappeared after changing to Detremin oil. Another patient experienced nausea and diarrhea while taking Detremin and was changed to Divisun, which was well tolerated.

## Discussion

Here we show that vitamin D_3_ supplementation of 1500–1600 IU/day for 1 year resulted in decreased antibiotic consumption. The decreased antibiotic consumption could almost solely be explained by the decrease of antibiotics for treating respiratory tract infections. Vitamin D_3_ supplementation was also associated with increased odds of staying antibiotic-free during the year after vitamin D_3_ supplementation. These results are in line with data from previously performed clinical trials showing beneficial effects of vitamin D on respiratory tract infections [4, 10, 13, 17, 18], whereas the effects on other infections are less clear [21, 22].

Surprisingly, the subgroup analysis for the different vitamin D levels at inclusion showed that the patients with lowest levels at inclusion (25OHD levels < 30 nmol/L), had less benefit of vitamin D_3_ supplementation than patients with 25OHD levels > 50 nmol/L. This might be explained by the fact that half of the patients with levels < 30 nmol/L at baseline never reached above 75 nmol/L, which in previous studies have been shown to be the critical threshold level protection against RTIs [19, 20]. In addition, patients who actually reached above 75 nmol/L might have obtained these levels too late in order to experience any benefit with regard to RTIs in the current study-protocol. In contrast, patients with 25OHD levels >50 nmol/L at baseline reached median levels of 90 nmol/L and the majority of the patients reached 25OHD-levels above 75 nmol/L (Table 3). Notably, it is likely that vitamin D follows the sigmoid dose-response curve that is applicable for many nutrients. The best effect of supplementation occurs in the exponential phase of the curve; i.e. the most deficient individuals need more vitamin D to increase their serum-levels and those already replete have no additional effect of extra vitamin D [23].

Interestingly, patients on IgG supplementation had no further beneficial effect of vitamin D treatment. Probably, IgG supplementation is the most potent immunomodulatory treatment available for these patients and that additional immune-active interventions could not boost the immune system further.

The mechanism for how supplementation with vitamin D can protect the respiratory tract against infections has been intensely studied during recent years [24]. Data from experimental trials suggest that vitamin D activates the innate immune system via upregulation of endogenous peptide antibiotics [1]. The most studied antimicrobial peptide in this respect is the cathelicidin LL-37, which expression is under direct control of the vitamin D receptor in macrophages and bronchial epithelial cells [25, 26]. LL-37 has potent antimicrobial activities against many respiratory pathogens, including *Streptococcus pneumoniae*, *Haemophilus influenza*, *Moraxella catharralis* and influenza-virus [27–30]. In addition to boosting effects on the innate immune system, vitamin D also modulates T-cell responses and generally dampens excessive inflammation by down-regulating pro-inflammatory cytokines [24]. Thus, there is a solid rationale based on experimental data how vitamin D supplementation could prevent RTI’s.

The main limitation of this study is the lack of a non-supplemented control-group. However, for practical reasons it was not possible to have such a group. Instead, we designed the study using every patient as its own control, which enabled a comparison of 12 months before supplementation with the following 12 months with supplementation. It could be argued that the observed effect could be explained by a changed policy on antibiotic stewardship and that all patients had fewer antibiotic prescriptions regardless of vitamin D_3_ supplementation. In fact, the number of antibiotic prescriptions has decreased significantly during the last five years in Sweden and the effect is greatest for respiratory antibiotics in children (www.folkhalsomyndigheten.se). In adults, however, the effect is not as evident (www.folkhalsomyndigheten.se). To control for this potential bias, we obtained information on the antibiotic prescription for the whole Immunodeficiency Unit from an independent source (www.strama.se). In fact, the antibiotic prescriptions at our Unit did not decrease (38 prescriptions/month before and 40 prescriptions/months after). Thus, despite the lack of a proper control group we have failed to find other obvious reasons to our observation that vitamin D_3_ supplementation could be beneficial in this group of selected patients with increased susceptibility to RTIs.

Another limitation with this study is that we only had access to antibiotics prescribed and purchased in Sweden. However, most patients are well connected to our unit and we think that the potential loss of information on antibiotics prescribed outside Sweden has a minor impact on the results.

In addition, we lack information whether the patients really have taken their antibiotics every day as prescribed. A prescription of antibiotics might not always reflect a true infection and–reciprocally—a true bacterial infection might not always results in an antibiotic prescription. However, we believe that the endpoint in this study is a relevant and non-biased parameter, since it is not affected by patient-related factors or subjective opinions. In fact, we have not been able to find any similar approach where an intervention with vitamin D_3_ supplementation is connected to unbiased antibiotic prescriptions obtained from a national registry.

Importantly, there were no serious adverse events reported and vitamin D supplementation was well tolerated. This indicates that vitamin D_3_ supplementation of 1500–1600 IE /day for 1 year is safe to use in immunodeficient patients with 25-OHD levels less than 75 nmol/L. The safety on vitamin D_3_ supplementation has been debated and it has been suggested that a cautionary approach should be adopted due to possible long term negative effects on mortality [31]. This assumption is mainly based on a large cohort study in Denmark, where a J-shaped curve was found, suggesting that not only very low vitamin D levels, but also supra-physiological levels were associated with an increased mortality [32]. In contrast, a large Cochrane analysis comprising 159 randomized trials that compared any type of vitamin D in any dose with any duration and route of administration versus placebo showed that vitamin D_3_ (but not D_2_) decreased mortality [33]. Interestingly, two recent studies clearly showed that vitamin D could be given safely to pregnant women in doses from 2400–4400 IU/day [34, 35]. In these two recent RCTs, no adverse events connected to the study drug could be observed. Thus, today there is no data suggesting that vitamin D supplementation in doses between 2000–4000 IU/day would confer any risks for patients.

The clinical implications of our findings are potentially important. It seems possible that vitamin D supplementation can reduce the number of antibiotic prescriptions and the number of RTI’s [4, 5]. This is of course beneficial for the individual patient with regard to quality of life, but also for the society given the significant economic cost connected to sick leaves due to RTI’s. A study from Norway, with a similar insurance system to Sweden, estimated the direct costs for one influenza season to 22 million USD and–more importantly–the number of lost working days to 793.000, leading to a productivity loss of 231 million USD [36]. Given this large economic burden connected to influenza alone, any reduction of these figures would cumulatively lead to large savings for the society.

In conclusion, our findings indicate that vitamin D_3_ supplementation of 1500–1600 IU /day is safe to use in immunodeficient patients with 25-OHD levels less than 75 nmol/L; it decreases antibiotic consumption in patients without immunoglobulin substitution and seems to protect patients from respiratory tract infections.
